# In situ thrombosis in pulmonary arterial aneurysms due to Behçet’s disease and efficacy of ımmunosuppressive therapy

**DOI:** 10.1186/2049-6958-7-33

**Published:** 2012-10-18

**Authors:** Sevket Ozkaya, Unal Sahin, Aziz Gumus, Filiz Taşçı, Halit Çınarka, Asiye Yavuz

**Affiliations:** 1 RizeUniversity, Faculty of Medicine, Department of Pulmonary Medicine, Rize, Turkey; 2 RizeEducation and Research Hospital, Department of Radiology, Rize, Turkey

**Keywords:** Behçet’s disease, Pulmonary artery aneurysm, In situ thrombosis, Immunosuppressive treatment

## Abstract

BehçetDisease (BD) is a systemic vasculitis characterized by recurrent oral and genital ulcers and uveitis, arthritis, and involvement of the gastrointestinal tract, central nervous system and blood vessels. The aneurysms of the pulmonary arteries, with or without thrombosis, are typical manifestation of BD. We report a case with BD, pulmonary arterial aneurysms(PAA) and in situ thrombosis. We aimed to show the effectiveness of immunosuppressive treatment on in situ thrombosis in a case with PAA and BD.

## Background

Behçet’s disease (BD) was firstly described by Hulusi Behçet in 1937. It is a systemic vasculitis characterized by recurrent oral and genital ulcers and uveitis, arthritis, and involvement of the gastrointestinal tract, central nervous system and blood vessels [1]. Pulmonary artery aneurysm (PAA) is reported in 1.5 % of adults with BD. Thrombosis of the pulmonary arteries in BD is usually an in situ thrombosis [2]. Some articles reported that immunosuppressive therapy is essential, and anticoagulant therapy might not be required for the treatment of venous disease associated with BD [3]. We aimed to show the effectiveness of immunosuppressive treatment on in situ thrombosis in a case with PAA and BD.

## Case presentation

**Figure 1:**
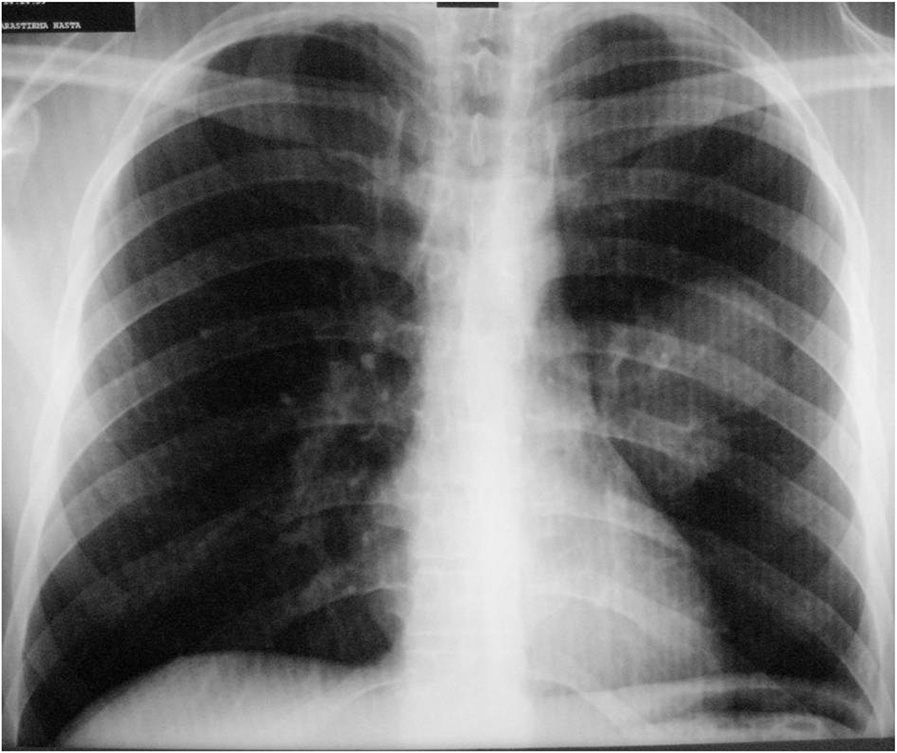
Chest radiography is showing the well-shaped, round hilar opacities on left hemithorax.

**Figure 2:**
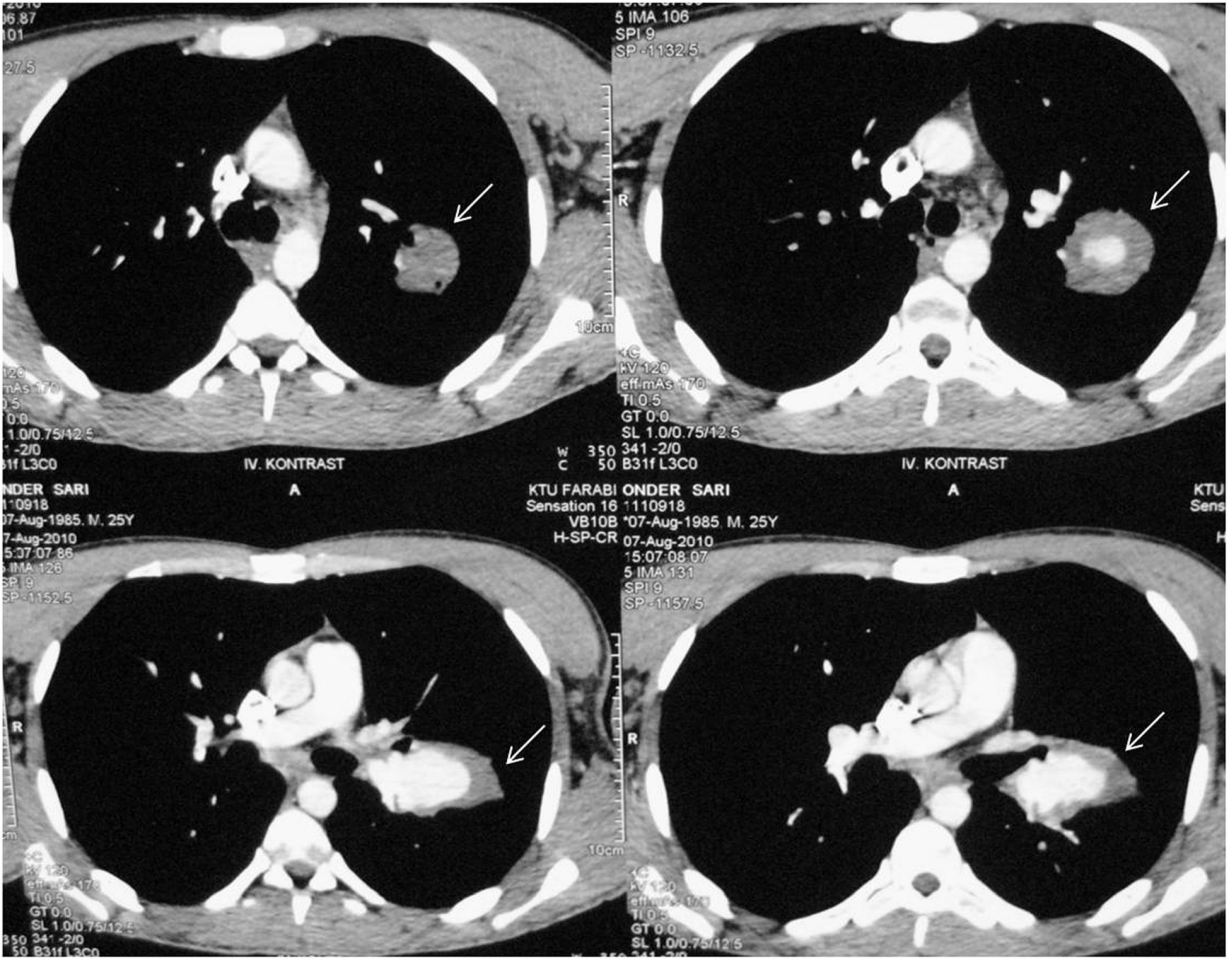
Thorax CT scans are showing the aneurysmatic dilatations and in situ thrombosis in the left pulmonary artery (white arrows).

**Figure 3:**
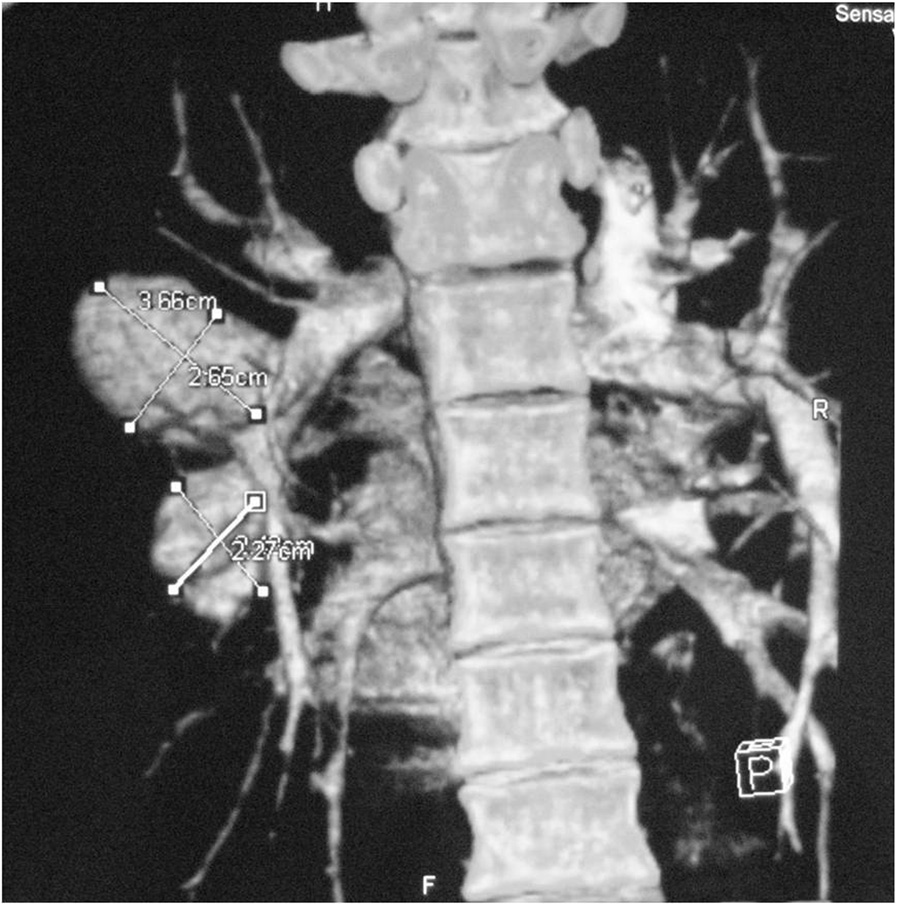
The thoracic MRI is showing the well-shaped, round hilar opacities on left hemithorax.

**Figure 4:**
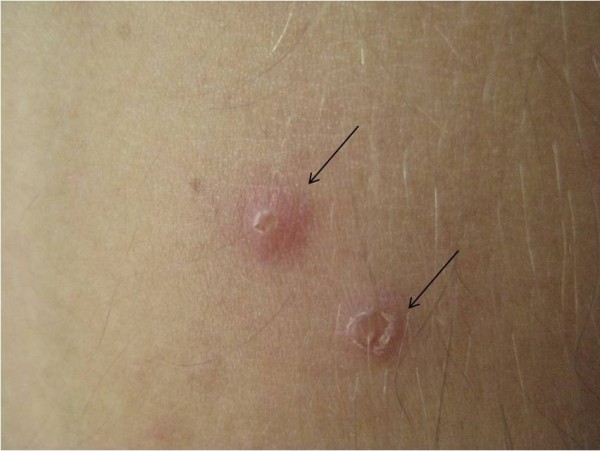
The positive pathergy test is seen with pustular lesion on injection area of body (black arrows).

**Figure 5:**
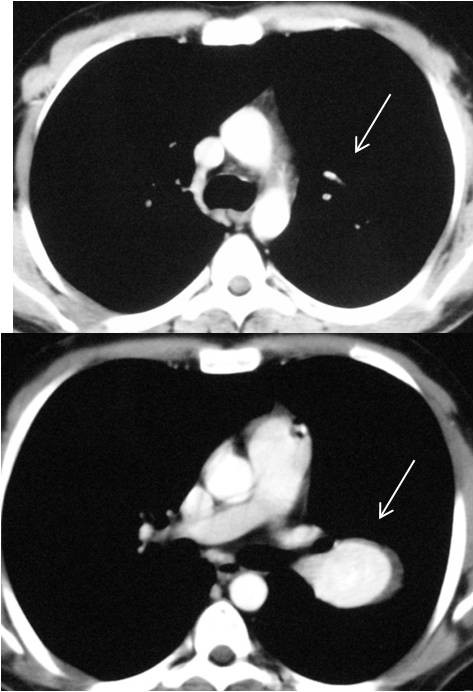
Thorax CT scans showing that in situ thrombosis in PAAs was completely resolved with immunsuppressive treatment (white arrows).

A 25-year-old,non-smoker turkish man was admitted to hospital with complaints of chest pain and cough. The chest radiography showed well-defined, rounded opacities on the left hemithorax (Figure 1). The thorax CT and MRI revealed vascular aneurysms with in situ thrombosis on the left pulmonary artery (Figure 2 and 3). The patient had history of recurrent oral and genital aphthous ulcers. The skin pathergy test was positive (Figure 4). BD was diagnosed based on these findings. The treatment was started with intravenous pulse methylprednisolone (250 mg per day for 3 days) and monthly 1,000 mg cyclophosphamide, and followed by 1 mg/kg per day of methylprednisolone orally, colchium and intravenous pulse of 1,000 mg cyclophosphamidemonthly. Thorax CT was repeated after 2 months of treatment. It showed that PAAs were reduced and in situ thrombosis in PAAs was completely resolved with immunosuppressive treatment (Figure 5). The approval of patient and of institution were taken to use their records for our study.

## Conclusions

After the aorta, the pulmonary arteries are the most common site of arterial involvement among the pulmonary manifestations in patients with BD [4]. PAAs associated with BD tend to be multiple, as seen in our patient. The hemoptysis is the commonest symptom of PAA in BD, and one of the leading causes of death [5]. In the present case there was no hemoptysis. The aneurysms of the pulmonary arteries, with or without thrombosis, are typical manifestation of BD [6]. Tunacı et al. reported mural thrombotic changes during regression of PAAs [7]. The underlying pathophysiologic process is inflammation of the *vasa vasorum* of the tunica media, which causes destruction of the elastic fibers of the media and dilatation of the vessel lumen. Thickening of the vessel wall is caused by inflammation and infiltration by lymphocytes, plasma cells and neutrophils. Thrombosis of the pulmonary arteries in BD is usually an in situ thrombosis [2,8]. Because the main problem is the inflammation of pulmonary arteries, the main stay of treatment should be the anti-inflammatory and immunosuppressive agents in patients with PAA and BD. A combination of cyclophosphamide and methylprednisolone is used most frequently in patients with PAA [9]. We know that anticoagulant therapy could increase the risk of aneurysmal rupture and anticoagulant drugs might be unnecessary in BD [5]. Mehta et al. reported a case with in situ thrombosis with BD [10]. They reported the patient had remained clinically stable with no further episodes of hemoptysis with immunosuppressive treatment including dexamethasone and cyclophosphamide [10]. However, there was no radiologically demonstrated efficacy of immunosuppressive treatment on in situ thrombosis. The aim of this report has been to demonstrate the effectiveness of immunosuppressive treatment on in situ thrombosis with PAA in a patient with BD. Contrast-enhanced thorax CT revealed the in situ thrombosis on the wall of PAAs. After the immunosuppressive treatment the in situ thrombosis was completely resolved and PAAs were reduced.

In conclusion, inflammation in pulmonary arteries is causing in situ thrombosis and it contributes to the development of PAAs in patients with BD. The anti-inflammatory and immunosuppressive drugs are essential for the treatment of in situ thrombosis and PAAs in patients with BD.

## Consent

Written informed consent was obtained from the patient for publication of this Case report and any accompanying images. A copy of the written consent is available for review by the Editor-in-Chief of this journal.

## Competing interest

The authors declare that they have no competing interests.

## Authors’ contributions

SO, US, AG, FT, HC, AY have made substantial contributions to conception and design, or acquisition of data, or analysis and interpretation of data. All authors read and approved the final manuscript.
